# Radiomic Phenotypes for Improving Early Prediction of Survival in Stage III Non-Small Cell Lung Cancer Adenocarcinoma after Chemoradiation

**DOI:** 10.3390/cancers14030700

**Published:** 2022-01-29

**Authors:** José Marcio Luna, Andrew R. Barsky, Russell T. Shinohara, Leonid Roshkovan, Michelle Hershman, Alexandra D. Dreyfuss, Hannah Horng, Carolyn Lou, Peter B. Noël, Keith A. Cengel, Sharyn Katz, Eric S. Diffenderfer, Despina Kontos

**Affiliations:** 1 Center for Biomedical Image Computing and Analytics, University of Pennsylvania, Philadelphia, PA 19104, USA; jose.luna@wustl.edu (J.M.L.); rshi@mail.med.upenn.edu (R.T.S.); 2 Department of Radiology, University of Pennsylvania, Philadelphia, PA 19103, USA; leonid.roshkovan@pennmedicine.upenn.edu (L.R.); michelle.hershman@pennmedicine.upenn.edu (M.H.); peter.noel@pennmedicine.upenn.edu (P.B.N.); sharyn.katz@pennmedicine.upenn.edu (S.K.); 3 Mallinckrodt Institute of Radiology, Washington University in Saint Louis, Saint Louis, MO 63110, USA; 4 Department of Radiation Oncology, University of Pennsylvania, Philadelphia, PA 19104, USA; andrew.barsky@baptisthealth.net (A.R.B.); dreyfusa@mskcc.org (A.D.D.); keith.cengel@pennmedicine.upenn.edu (K.A.C.); eric.diffenderfer@pennmedicine.upenn.edu (E.S.D.); 5 Penn Statistics in Imaging and Visualization Center, Department of Biostatistics, Epidemiology, and Informatics, University of Pennsylvania, Philadelphia, PA 19104, USA; louc@pennmedicine.upenn.edu; 6 Department of Bioengineering, University of Pennsylvania, Philadelphia, PA 19104, USA; hhorng@seas.upenn.edu

**Keywords:** overall survival, ComBat, radiomics, non-small cell lung cancer, computed tomography

## Abstract

**Simple Summary:**

Personalized therapy of non-small cell lung cancer (NSCLC) relies heavily on histopathological analyses that require invasive biopsies that have relatively high costs, provide limited assessment of tumor heterogeneity and are associated with potentially life-threatening complications. This retrospective study is aimed at evaluating the potential benefit of using predictive models that integrate radiomic features extracted from computed tomography (CT) images and commonly assessed clinical predictors to characterize the overall survival (OS) of stage III NSCLC adenocarcinoma patients receiving chemoradiation. Different than previous studies, our proposed approach explicitly accounts for CT parameter heterogeneity, such as presence or lack of intravenous contrast material and differences in CT scanner vendors through feature harmonization. Using a relatively homogeneous population of 110 patients, our results demonstrate that radiomic biomarkers derived using feature harmonization significantly improved the prediction of OS in our cohort when combined with Eastern Cooperative Oncology Group (ECOG) status and age at diagnosis, suggesting their potential in assisting clinical decision making.) upon the baseline model (C-score = 0.65, CI = (0.57, 0.73)). Our results suggest that harmonized radiomic phenotypes can significantly improve OS prediction in stage III NSCLC after chemoradiation.

**Abstract:**

We evaluate radiomic phenotypes derived from CT scans as early predictors of overall survival (OS) after chemoradiation in stage III primary lung adenocarcinoma. We retrospectively analyzed 110 thoracic CT scans acquired between April 2012−October 2018. Patients received a median radiation dose of 66.6 Gy at 1.8 Gy/fraction delivered with proton (55.5%) and photon (44.5%) beam treatment, as well as concurrent chemotherapy (89%) with carboplatin-based (55.5%) and cisplatin-based (36.4%) doublets. A total of 56 death events were recorded. Using manual tumor segmentations, 107 radiomic features were extracted. Feature harmonization using ComBat was performed to mitigate image heterogeneity due to the presence or lack of intravenous contrast material and variability in CT scanner vendors. A binary radiomic phenotype to predict OS was derived through the unsupervised hierarchical clustering of the first principal components explaining 85% of the variance of the radiomic features. C-scores and likelihood ratio tests (LRT) were used to compare the performance of a baseline Cox model based on ECOG status and age, with a model integrating the radiomic phenotype with such clinical predictors. The model integrating the radiomic phenotype (C-score = 0.69, 95% CI = (0.62, 0.77)) significantly improved (p<0.005) upon the baseline model (C-score = 0.65, CI = (0.57, 0.73)). Our results suggest that harmonized radiomic phenotypes can significantly improve OS prediction in stage III NSCLC after chemoradiation.

## 1. Introduction

Lung cancer is the most common cause of cancer-related deaths in the United States [1], with over 135,000 lung cancer-related deaths estimated in 2020 [2]. Non-small cell lung cancer (NSCLC) represents over 80% of lung cancer cases [3], with the most common subtype being adenocarcinoma [4]. Within adenocarcinoma, numerous additional subtypes have been identified as novel mutation and biomarker testing has become available, allowing personalization of treatment options [5]. Even with increasing molecular personalization of therapies, data regarding predicted outcomes of therapies remain limited. Stage III NSCLC includes a group of patients with variations in localization and extent of the disease, with treatment approaches that have been enhanced with the inclusion of more active chemotherapy agents [6,7,8,9,10,11], refinements of radiation techniques [12,13,14] and the recent integration of immunotherapy [15,16,17,18,19]. However, data supporting current treatment approaches are based on heterogeneous populations with limited follow-up duration and inadequate statistical power [20,21]. In this paper, we propose a machine learning approach based on imaging phenotypes aimed at predicting the overall survival (OS) of stage III NSCLC adenocarcinoma patients receiving chemoradiation, to establish novel and relevant predictive biomarkers of treatment response with potential applications in lung cancer management.

The development of accurate models to predict disease recurrence in patients with NSCLC treated with definitive chemoradiotherapy, also known as treatment failure, could play a fundamental role in the efficient personalization of treatment. In fact, previous investigators have utilized clinicopathological data to develop predictive models for treatment failures, demonstrating an ability to predict patterns of failure in NSCLC [22] and to identify factors associated with increased or decreased risks of treatment failure [23]. However, tumoral molecular characterization and genomic analysis require tumor biopsies that are invasive and can be associated with life-threatening complications [24]. The applicability of such approaches to predict NSCLC response to treatment is limited by high costs and a long turnaround time, as well as the limited capture of tumor heterogeneity, a factor that is known to contribute to poorer patient outcomes in multiple types of cancer, such as NSCLC [25,26,27]. Routinely obtained lung images, such as pretreatment PET/CT scans, are a proven noninvasive method to study tumor physiology [28]. There is also evidence that somatic mutations drive distinct lung radiographic phenotypes that can be decoded through radiomics [29].

Standard of care medical images acquired during lung cancer management has a yet untapped wealth of information of in situ tumor architecture, heterogeneity and peritumoral environment, which may have prognostic implications. Radiomics, a quantitative method for assessing diagnostic images, involves detailed mathematical analyses of the images to determine whether or not particular patterns are observed in the data, which could, optimally, be used to assist in predictive analytics and clinical decision making [30]. In NSCLC, a CT scan of the chest is part of the standard workup of the disease, as well as a critical aspect of the radiotherapy treatment planning simulation process [5]. Given that essentially all patients with stage III NSCLC should have pre-treatment chest CT scans, these images may represent opportune targets for the radiomic analysis of predictors of local, regional, and distant failure, and/or OS in this population.

Multiple prior investigators have assessed the use of radiomics in developing predictive models for NSCLC disease control outcomes. Among the most recent related works is the one performed by Wang et al. [31] in which the authors developed a pipeline for the prediction of recurrence-free survival in resected stage I NSCLC with a mixed population of adenocarcinoma and squamous carcinoma using CT scans. Their results showed that radiomic features improved the predictive performance of the histology. Furthermore, Arshad et al. [32] assessed the predictive performance of radiomics performed on pre-treatment FDG-PET scans with images acquired with a variety of PET/CT scanners in a multicenter study of a population of mixed stage I–III NSCLC and different histologies. The obtained feature vector was able to predict OS using a validation set. In another study, Lee et al. [33] developed a pipeline for OS in a population of NSCLC adenocarcinoma with a variety of stages, and adjuvant therapies. Their results, obtained through cross-validation, showed that the predictive performance of a model based on clinicopathological variables improved when such variables are integrated with radiomic features. In another publication, van Timmeren et al. [34] carried out a study using longitudinal radiomic features extracted from cone beam CT (CBCT) scans using three validation sets. Their results showed that radiomic features extracted from CBCT scans did not seem to improve prognostication information possibly due to the heterogeneity within and between datasets. While there were differences in study design and patient cohorts between the studies (stage I NSCLC vs. all NSCLC stages; pre-treatment CT vs. pre-treatment PET/CT vs. on-treatment CBCT-based; radiotherapy vs. chemoradiotherapy vs. surgery as primary intervention), the studies were largely positive in demonstrating the ability of radiomics to predict recurrence-free survival or OS [31,32,33,34,35]. Even with these findings, some of these studies may have been limited by cohort heterogeneity, differences in imaging vendors, and the presence or absence of intravenous contrast material.

In this study, we investigate the association between OS and radiomic features extracted from chest CT scans of a relatively homogeneous cohort of stage III NSCLC adenocarcinoma patients at our institution using a predictive pipeline that explicitly accounts for image heterogeneity due to image acquisition protocols, vendors and intravenous contrast material.

## 2. Materials and Methods

### 2.1. Patient Cohort

CT scans were retrospectively analyzed from a set of stage III NSCLC patients previously treated at the University of Pennsylvania. This study was compliant with the Health Insurance Portability and Accountability Act, approved by our Institutional Review Board, and in accordance with U.S. Common Rule (Penn IRB protocol #832329). The inclusion criteria were limited to several parameters: (1) histologically confirmed AJCC 7th Edition stage III NSCLC adenocarcinomas, (2) pre-treatment CT scans within 2 weeks of chemoradiation, and (3) first radiation course to primary tumor in the lung. Patients with pre-operative radiotherapy as well as patients with post-operative CT scans were excluded. An initial cohort of 132 consecutive patients treated from April 2012 through October 2018 at our institution was identified for this study. From the initial cohort, 14 patients were excluded due to incomplete or corrupted imaging files, and 3 more patients were ruled out due to images with collapsed lungs or mediastinal primary tumors that would not allow for precise segmentation. Finally, 5 more patients whose treatment was interrupted early because of death or other adverse events were also excluded from the study. Therefore, a total of 110 patients diagnosed with stage III NSCLC adenocarcinoma patients, with available CT scans and consistently treated with chemoradiation, were included in our analysis. All patients were treated with concurrent or sequential chemoradiation with platinum containing regimens, and either proton beam therapy (PBT) or intensity-modulated radiation treatment (IMRT).

### 2.2. Radiomic Feature Extraction

For each patient, the primary tumor was manually segmented by one out of three experienced radiologists (S.K. 21 yr., L.R. 9 yr. and M.H. 7 yr.) from a pretreatment, preoperative CT scan. Images were preprocessed using histogram normalization based on z-scores to compensate for outliers due to artifacts within the image. Additionally, since 65% of the studies have slice thickness of 3 mm, an isotropic spline interpolation to 3 mm was applied to all the images to account for possible differences in image spatial resolution. A set of 107 radiomic features was extracted using the software Pyradiomics 3.0.1 [36], including morphologic features closely related to tumor volume and shape irregularity, such as area, sphericity, compactness, elongation and flatness, usually associated with disease progression [37]. We also included radiomic features capturing structural, co-occurrence matrix, gray-level size zone matrix textures, run-length, and first order features; all extracted from the primary tumor. Briefly, structural features depict intensity variations between central and neighboring voxels. Co-occurrence features examine the spatial distribution of voxel intensity values by extracting frequency information of gray-level intensity values within a neighborhood of voxels in a specific linear orientation. Gray-level size zone features describe the connectedness of varying intensity levels within an image. Run-length features capture the coarseness of an image in specific linear directions. First-order features assess the distribution of gray-level voxel intensities within an image.

### 2.3. Clinical Features

In this study, we collected a set of continuous and categorical predictors of OS reported in the existing literature. The continuous features were age at diagnosis [38], body mass index (BMI) [39], and smoking pack per year [40]. The categorical features were race [41], gender [38], and Eastern Cooperative Oncology Group (ECOG) performance status at diagnosis [42].

### 2.4. ComBat Harmonization

ComBat is a harmonization technique originally developed for genomic studies [43], which has been shown to be effective for correcting statistical variation due to batch effects while protecting known associations with covariates of interest by using an empirical Bayesian framework. ComBat has recently been incorporated in radiomic analysis and has been able to correct variation in radiomic features due to batch effects, such as image acquisition protocols across different clinical practices [44,45,46,47,48,49,50,51,52], using empirical Bayes to estimate location and scale parameters to alleviate such variation. We used a nested ComBat approach developed at our center [53] to simultaneously correct for multiple batch effects of interest in CT acquisition. The nested approach was initialized with the original radiomic features being z-scored first as the input data, and at each iteration the radiomic features were separately harmonized by all batch effects, namely, CT contrast (i.e., a categorical variable with values contrast/non-contrast enhanced) and CT scanner vendors (i.e., a categorical variable with values Philips Healthcare (Best, The Netherlands)/Siemens Healthineers (Erlangen, Germany)/GE Healthcare (Chicago, IL, USA)). The resulting harmonized feature sets were assessed for significant differences between distributions across batches due to the corresponding batch effect using the Kolmogorov–Smirnov test at a two-sided *p*-value significance level α=0.05. The harmonized feature set with the lowest number of features with detected differences in distribution was selected as the input data for the next iteration, with its corresponding batch effect removed from the list. This procedure was repeated until there were no batch effects remaining on the list, with a single feature set sequentially harmonized by all batch effects as the output. The neurocomBat package [54] for R 4.0.2 [55] was used as a basis in this nested implementation of ComBat.

### 2.5. Unsupervised Hierarchical Clustering

We used principal component analysis to reduce the dimensionality of the ComBat harmonized features by selecting the first principal components (PCs) explaining 85% of the variance of the features. After that, we performed unsupervised hierarchical clustering, applying the maximum distance linkage. Consensus clustering was used to identify the optimal number of clusters (n=2), to represent high and low risk of death in our cohort. For each phenotype cluster, we assessed the differences across radiomic phenotypes of the clinical covariates. For continuous features, i.e., age at diagnosis, BMI, and smoking pack per year, we used a Kruskal–Wallis test, and for the categorical features, i.e., race, gender and ECOG status, we used a chi-squared test at a two-sided *p*-value with α=0.05.

### 2.6. Univariate Analysis

The individual predictive performance of each clinical variable, as well as the imaging phenotype based on harmonized (i.e., Phenotype (ComBat)) and non-harmonized (i.e., Phenotype (non-ComBat)) features, were assessed by fitting a Cox proportional hazards regression model and then estimating the C-score associated with the fitted models. The 95% confidence interval of the average C-score was calculated using a total of 50,000 bootstrap replicates assuming the standard t-distribution-based approximation. All the analyses were implemented using the SimpleITK [56], SciPy [57], scikit-learn [58] and lifelines [59] libraries for Python 3.7.6 [60], as well as the robustHD [61], gplots [62] and sigclust packages [63] for R 4.0.2 [55].

### 2.7. Multivariate Analysis

Combinations of subsets of clinical features were used to fit several Cox proportional hazards model to assess their combined predictive performance. The Cox model, which combined the clinical features with the best performance, was then selected as the baseline model. In addition, to assess the predictive performance of the radiomic phenotypes, we fitted Cox proportional hazards models combining the clinical features of the baseline models with Phenotype (ComBat) and Phenotype (non-ComBat). We also compared the predictive performance of the model combining PCs to the baseline variables, but only considered the first PCs whose hazard ratios (HR) were significantly different from 1 in the corresponding Cox proportional hazards model to reduce overfitting.

This analysis corresponds to the development and validation of a predictive model using resampling or analysis type 1b as specified by Collins et al. in [64]. Finally, we compared the predictive performance of each model to separate the two groups, namely high and low risk of death, by calculating their respective C-score.

**Figure 1:**
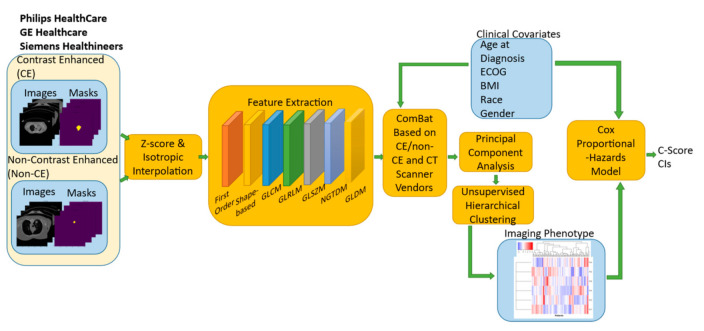
Pipeline for multivariate survival analysis. Notice the ComBat-based harmonization block right after the feature extraction that accounts for batch effects due to differences in CT scan vendors and the presence or absence of intravenous contrast.

As in the univariate analysis, 95% confidence intervals of the average performance measurements were calculated using estimates as bootstrapped samples using the standard t-distribution-based approximation with 50,000 bootstrap replicates. Our proposed pipeline for multivariate survival analysis in stage III NSCLC adenocarcinoma is summarized in Figure 1. Given the modest size of our current cohort, we also fitted non-cross-validated Kaplan–Meier curves aimed at illustrating the improvement in predictive performance of the baseline model based on clinical variables and the model integrating clinical variables and imaging phenotypes.

## 3. Results

### 3.1. Patient Characteristics

The characteristics of the 110 patients in the study are provided in Table 1 and Table 2. The patients’ median age was 66 years (range 60–71). Patients received a median radiation dose of 66.6 Gy, at 1.8 Gy per fraction (range 60.0–66.7 at 1.8 Gy per fraction). Within our cohort, 56 events of death (50.9%) were registered during the duration of the study. Median follow-up time was 24.2 months (12.4–35.1 month range), with a median OS of 30.6 months, 1 year OS of 79.8%, 2 year OS of 59.8%, and 5 year OS of 35.9%. Overall, 89% of the patients received concurrent chemotherapy, with carboplatin-based doublet combination (55.5%) being the most common regimen, followed by cisplatin-based doublet (36.4%). Radiation was delivered with proton beam treatment (55.5%) and photon beam treatment (44.5%).

### 3.2. Univariate Analysis

**Figure 2:**
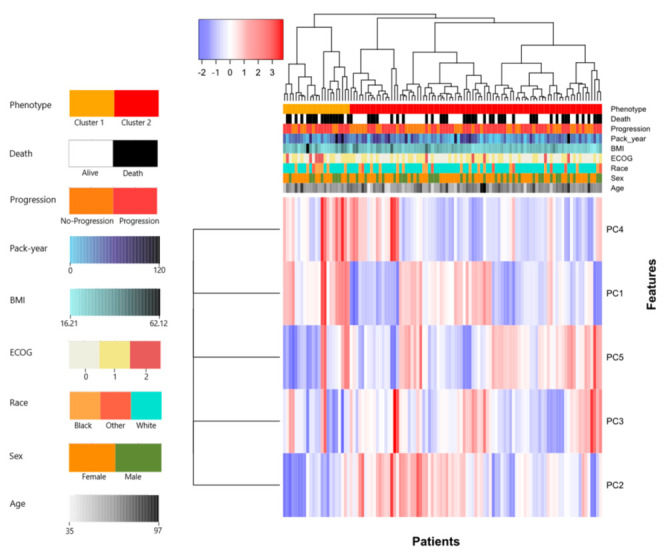
Heatmap representing the imaging phenotype calculated through unsupervised hierarchical clustering. The imaging phenotype obtained from harmonized features using ComBat is able to predict the event of death (p=0.025) and ECOG status (p=0.048 ).

In univariate analysis, the ECOG status with C-score = 0.62 (95% CI (0.55, 0.69)), is the only clinical feature able to predict systematic survival differences in our cohort. Additionally, Phenotype (ComBat) with C-score = 0.61 (95% CI (0.54, 0.67)) shows good predictive performance. A detailed heatmap showing the resulting phenotype labels across the patient cohort, as well as its qualitative associations with clinical covariates (i.e., pack year, BMI, ECOG, race, sex, age) and outcomes (i.e., death and progression) is shown in Figure 2. Furthermore, as suggested by Figure 2, Phenotype (ComBat) is calculated using the first five PCs of the radiomic features since they explain 85% of their variance. Moreover, based on chi-square test results, Phenotype (ComBat) is able to predict the event of death (p=0.025) and the ECOG status (p=0.048), which complies with the distribution of values indicated for the event of death and ECOG status with respect to the phenotype shown in Figure 2. More details about the predictive performance of Phenotype (ComBat) are provided in Figures S1 and S2 and Table S1. Moreover, the results of univariate analysis are summarized in Table 3.

### 3.3. Multivariate Analysis

In multivariate analysis, after assessing different subsets of the available clinical variables, i.e., age at diagnosis, BMI, pack per year, race, gender, and ECOG status, the results suggest that the model integrating age at diagnosis and ECOG status (i.e., ECOG + Age) showed the best predictive performance with C-score = 0.65 (95% CI (0.57, 0.73)), therefore such model was used as the baseline model for comparison. A predictive model integrating such clinical variables with the non-harmonized imaging phenotype (i.e., Phenotype (non-ComBat) + ECOG + Age) did not exhibit a significant improvement (p=0.15) with C-score = 0.66 (95% CI (0.58,0.74)) with respect to the baseline model. However, a significant improvement (p=0.003) is seen when such clinical variables are integrated to the imaging phenotype obtained from harmonized features using ComBat (i.e., Phenotype (ComBat) + ECOG + Age) with C-score = 0.69 (95% CI (0.62, 0.77)). Finally, the integration of the first PCs whose hazard ratios were significantly different from one in the Cox model, narrowed the number of PCs down to the first two, with the second PC exhibiting HR=0.94,p=0.03. The model integrating the first two PCs with the clinical features (i.e., 2 PCs + ECOG + Age) did not significantly improve the performance of the baseline model either (p=0.27), with C-score = 0.65 (95% CI (0.60, 0.75)). The results of the multivariate analysis are summarized in Table 4. The non-cross-validated Kaplan–Meier curves comparing the performance of the baseline model based on clinical variables and the model integrating clinical variables and imaging phenotypes are shown in Figure S3a,b.

## 4. Discussion

In this paper, we report our findings of the use of radiomic features to predict OS using pre-treatment chest CT scans from a relatively homogeneous cohort of stage III NSCLC adenocarcinoma patients treated with definitive chemoradiotherapy at our institution. We found that the proposed radiomic phenotype, based on unsupervised hierarchical clustering, was able to predict for OS, and significantly improve upon the predictive performance of well-known clinical predictors, such as ECOG status and age at diagnosis. Furthermore, the use of ComBat feature harmonization contributed favorably to the performance of the model.

On univariate analysis, we found that ECOG status (C-score = 0.62) and Phenotype (ComBat) (C-score = 0.61) were able to predict OS differences in our cohort. On multivariate analysis, we found that, while the model based on ECOG + Age (C-score = 0.65) and the model based on 2 PCs + ECOG + Age (C-score = 0.65) each demonstrated an ability to predict for OS, the predictive performance significantly improved further (p=0.003) with the addition of the radiomic phenotype with ComBat only, i.e., Phenotype (ComBat) + ECOG + Age (C-score = 0.69). Notice that, although these results are non-cross-validated, we are comparing the actual models using the baseline (ECOG + Age) model as a reference and assessing the improvements in predictive performance using LRT, which reassures our results, since the bias is toward conservatism.

Our findings suggest that the integration of the radiomic phenotype to our predictive model provides an additional discerning value in the ability to predict OS. Given the increased C-score with the addition of ComBat feature harmonization to the radiomic phenotype aspect of our predictive model, we argue that ComBat provides a substantial improvement over the existing model without the use of ComBat. Specifically, given that ComBat utilizes existing covariates to account for differences between CT scanner vendors, as well as between images based upon the presence or absence of intravenous contrast (of note, over 25.5% of scans in our cohort were performed with contrast), we conclude that the improvement in the predictive model is due to ComBat’s ability to add to the consistency of radiomic features across the various scans.

Prior reports of the use of ComBat in computational medical imaging to solve classification tasks have been published in the literature, mainly to harmonize images acquired in different clinical centers and under different acquisition protocols in classification problems. Regarding feature harmonization to account for batch effects due to multiple centers, Whitney et al. [44] used a set of dynamic contrast-enhanced magnetic resonance images (DCE-MRI) from centers in the United States and China to develop models able to classify benign from malignant lesions in breast cancer patients. Similarly, Lucia et al. [45] developed a pipeline able to classify binary outcomes of disease progression and loco-regional control using radiomic features derived from PET and MRI scans in patients with cervical cancer from four different centers located in France and Canada. Furthermore, Masson et al. [46] developed a radiomic pipeline able to predict non-response to induction chemotherapy in patients with laryngeal cancer treated at five different centers. In the specific case of lung cancer patients, which is the population of interest in our study, Dissaux et al. [47] carried out a multicenter study using PET/CT scans to predict local control in early stage NSCLC on patients receiving stereotactic body radiation therapy (SBRT) from three different centers. Moreover, Garau et al. [48] developed a model able to classify benign from malignant nodules of lung cancer patients using low dose CT (LDCT) scans from two different datasets. Different than our approach, the previously mentioned studies do not consider survival outcomes, but rather limit their analyses to classification problems, such as cancer detection or tumor classification as benign or malignant. Furthermore, the two related studies in lung cancer [47,48] do not target the population of stage III NSCLC adenocarcinoma patients treated with chemoradiation that we analyzed in our study. To the best of the authors’ knowledge, no previous studies regarding radiomic pipelines for survival outcomes in lung cancer have been reported in the literature.

Regarding the harmonization of features extracted using different image acquisition devices, Shayesteh et al. [49] implemented a pipeline to classify responders from non-responders using pre-treatment, post-treatment and delta radiomics extracted through two MRI scanners from a cohort of colorectal cancer patients treated with neoadjuvant chemoradiation. About survival outcomes, Hotta el al. [50] used a cohort of rectal cancer patients to predict OS and progression-free survival (PFS) using PET scans acquired using three different PET scanners. Similarly, Nakajo et al. [51] used ComBat to account for differences between two PET scanners to predict OS and PFS using a cohort of endometrial cancer patients. Finally, Crombé et al. [52] developed a pipeline to predict metastatic-relapse-free survival using ComBat to compensate variations across three different MRI scanners on a population of sarcoma patients.

While prior reports of the use of ComBat exist for breast cancer [44], colorectal cancer [49,50], gynecologic cancers [45,51], laryngeal cancer [46], and sarcoma [52], our report is one of the first to describe the use of ComBat to develop a predictive model for NSCLC treatment outcomes, and specifically, within a relatively homogeneous population consisting entirely of patients with stage III NSCLC adenocarcinoma treated with definitive chemoradiotherapy with similar treatment regimens. Prior studies have utilized ComBat to generate a predictive model in low-dose CT screening for lung cancer diagnosis [48] and for pre-treatment PET/CT prediction of stereotactic body radiotherapy outcomes in early stage NSCLC [47], but none to date have explicitly assessed OS in a stage-III-only NSCLC population.

The previous literature on the implementations of ComBat to harmonize radiomic features suggests that the main causes of batch effects in image datasets are mainly differences in image acquisition protocols, as well as device-associated factors (e.g., peak kilovoltage (kVp), convolutional kernels, scanner model and manufacturer) [44,45,46,47,48,49,50,51,52]. However, correcting for batch effects considering such a variety of variables can be difficult to manage, since depending on the nature of the covariates, the batch groups could become too small and unbalanced limiting the power of ComBat to accurately model the batch effect in a data-driven fashion. Therefore, the general approach is to model batch effects based on inclusive variables that are problem dependent, such as country of origin of the data (e.g., United States/China [44]), the clinical institution where the data was acquired (e.g., University Hospitals of Brest/Nantes/McGill [45]), or scanner manufacturers (e.g., Discovery 600 M/Discovery MI, both made by GE Healthcare [51]) in studies that use images acquired using different scanners. Furthermore, feature harmonization based on convolutional kernels used by the CT scanners is limited by their unbalanced distribution and modest sample sizes per kernel category, i.e., Philips (kernel C, n=67), Siemens (kernels B50f, n=13; B30f, n=8; I50f/3, n=8; I70f/3, n=3; B30s, n=2; B31s, n=1; B40f, n=1) and GE (kernel LUNG, n=7). Therefore, following previous studies, a categorical covariate with the three categorical values, namely, Philips, Siemens and GE, was implemented to encompass the effect of the convolutional kernels while allowing more balanced batches.

**Figure 3:**
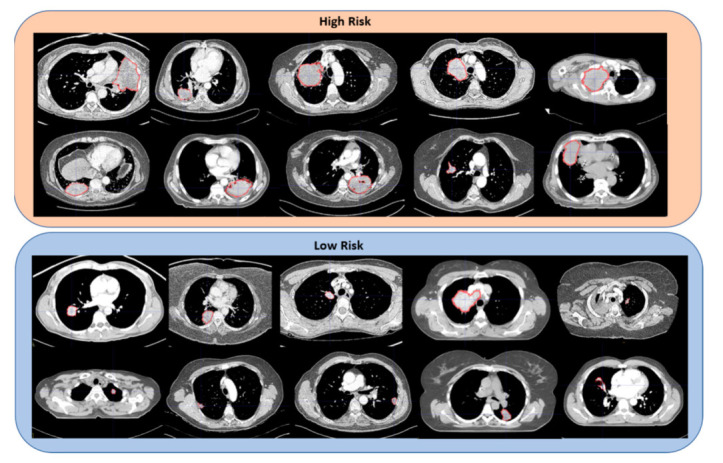
Sample of high- and low-risk patients classified by our predictive pipeline. Notice that the tumors in CT scans belonging to patients with a high risk of death tend to be larger than the ones in patients with a low risk of death.

**Figure 4:**
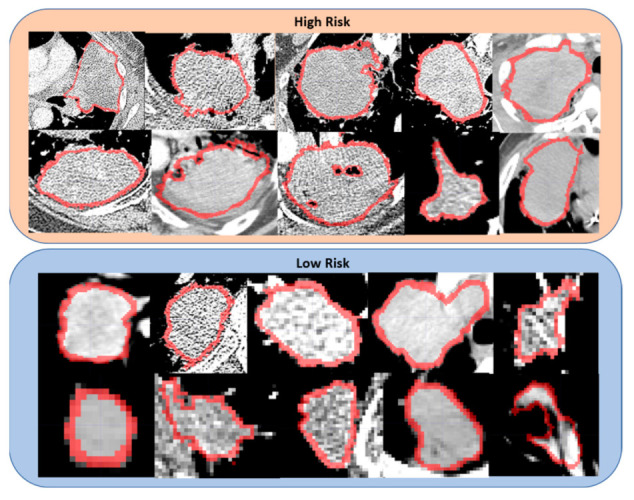
Details of the tumors classified by our predictive pipeline as high and low risk of death. Notice that the tumors in CT scans belonging to patients with a high risk of death tend to be more heterogeneous than the ones in patients with a low risk of death.

In Figure 3, we show a subset of CT scans classified by our proposed predictive pipeline as corresponding to patients with low or high risk of death. Notice that the two groups seem to be differentiated by the average tumor size, i.e., patients with high risk of death tend to have larger tumors than low risk patients. Furthermore, as shown in Figure 4, tumors of patients with high risk of death tend to be visually more heterogeneous than patients with low risk of death. Such heterogeneity can be related to the presence of tumor necrosis, which was shown to be a factor indicating more aggressive tumor behavior and was reported to contribute to poorer patient outcomes in multiple types of cancer, including NSCLC [25,26,27]. For the sake of completeness, we estimated the tumor volume through the mesh volume feature available in Pyradiomics. The C-score corresponding to the tumor volume alone was 0.60 (95% CI (0.51, 0.68)), which is significantly lower (p<0.005) than the C-score of Phenotype (ComBat) of 0.61 (95% CI (0.54, 0.67)) when predicting overall survival (see Figure S1c). Furthermore, when tumor volume is integrated with the baseline model, its performance is C-score = 0.67 (95% CI (0.59, 0.75)), which is significantly lower (p<0.005) than the model integrating Phenotype (ComBat) and clinical features with C-score = 0.69 (95% CI (0.62, 0.77)) (see Figure S3c). Notice that tumor volume is one of the radiomic features used to derive Phenotype (ComBat), which means that there is an association between these two predictors already, with Phenotype (ComBat) significantly outperforming tumor volume.

The proposed evaluation method is mainly based on conventional inferential statistics to calculate C-scores for survival prediction, as well as confidence intervals, which are not intrinsically high cost, especially given our modest cohort size (n=110). Furthermore, our approach relies on calculations based on highly optimized and numerically accurate implementations of algorithms, such as principal component analysis, hierarchical clustering, and batch effect correction using ComBat, currently available in standardized packages for python (e.g., SimpleITK, SciPy, scikit-learn, lifelines and neuroComBat) and R (e.g., robustHD, gplots and sigclust), which have been optimized to, among other things, reduce execution time. Finally, notice that the standardized packages used in this application are freely usable and distributable, therefore no additional financial cost has been added by our implementation.

A few limitations exist. First, most patients were treated prior to the initial publication of the PACIFIC trial in late 2017 [16], which in its most recent update showed a significant OS benefit to the addition of consolidative durvalumab in patients who underwent definitive concurrent chemoradiotherapy for stage III NSCLC [65], and therefore consolidative immunotherapy was not routinely administered in our cohort. As such, outcome estimates in our report may underestimate what outcomes may be in the present day with the potential addition of durvalumab. Next, seven patients in our cohort did not have specific details available regarding chemotherapy agents used or sequencing of chemotherapy with radiotherapy. Additionally, while the C-score performance of our model showed an encouraging degree of efficacy, it still has room for further refinements to better optimize its performance for potential use in the clinical setting. Furthermore, this study uses model baselines for comparison and no cross-validation/external testing was performed. Finally, the proposed method is specific to predict OS in stage III NSCLC adenocarcinoma patients as described in Section 2.1, and Table 1 and Table 2. However, it could be useful in new studies for other types of lung cancer as long as the hyperparameters of the algorithms are optimized and the cohorts are large enough so that they allow for stratification of patients according to well-justified scientific criteria. Further assessments of radiomic predictive modeling using ComBat in additional homogeneous cohorts will be valuable in further validating and improving upon the results shown in this report.

## 5. Conclusions

We report our results using radiomic features, utilizing ComBat feature harmonization, to predict OS using pre-treatment chest CT scans from a relatively homogeneous cohort of stage III NSCLC adenocarcinoma patients managed with definitive chemoradiotherapy. Our results suggest that imaging phenotypes extracted through unsupervised machine learning using ComBat-based feature harmonization can enhance the predictive performance of well-known clinical predictors of OS, which is particularly relevant to stage III NSCLC management, which in recent years has incorporated novel variants of chemotherapy, radiotherapy modalities and immunotherapies to its treatment. Importantly, the additional use and refinement of this model are critical to be able to incorporate predictive modeling safely and responsibly into the clinical decision making.

## Figures and Tables

**Table 1:** Categorial patient characteristics. Description of clinical characteristics of the cohort with their respective categorization and percentages.

Categorical Features	Classes	No. of Patients	(%)
Contrast Enhancement	Non-Contrast Enhanced	82	74.5
	Contrast Enhanced	28	25.5
CT Scanner Manufacturer	Philips Healthcare	67	60.9
	Siemens Healthineers	36	32.7
	GE Healthcare	7	6.4
Sex	Female	68	61.8
	Male	42	38.2
Race	White	80	72.7
	African American	22	20.0
	Asian	3	2.7
	Native American	1	0.9
	Other	4	3.6
Marital Status	Married	66	60.0
	Single	24	21.8
	Divorced	8	7.3
	Widowed	8	7.3
	Separated	4	3.6
Radiation Modality	Proton	61	55.5
	Linac	49	44.5
ECOG Status	0	50	45.5
	1	48	43.6
	2	10	9.1
	Unknown	2	1.8
Tobacco Use	Former Smoker	91	82.7
	Current Smoker	13	11.8
	Never Smoker	6	5.5
Histology	Adenocarcinoma	110	100.0
Chemotherapy Agents	Carboplatin-based Doublet	61	55.5
	Cisplatin-based Doublet	40	36.4
	Platinum-based Triplet	2	1.8
	Unknown	7	6.4
Chemotherapy	Concurrent	89	80.9
	Sequential	14	12.7
	Unknown	7	6.4

**Table 2:** Continuous patient characteristics. Description of continuous characteristics of the cohort with their respective median and interquartile ranges.

Continuous Features	Median	Range *
Age (yr.)	66	(60–71)
Radiation Dose Delivered (Gy)	66.6	(60.0–66.7)
Dose per Fraction (Gy)	1.8	(1.8–1.8)
BMI (kg/m^2^)	26.5	(23.8–29.9)
Pack per year (smokers only)	35.0	(20.0–50.0)

* Interquartile range.

**Table 3:** Univariate analysis results. ECOG status is the only clinical variable showing good predictive performance. Additionally, Phenotype (ComBat) is able to predict survival differences in our cohort.

Predictor	C-Score	95% CI
ECOG Status	0.62	(0.55, 0.69)
Phenotype (ComBat)	0.61	(0.54, 0.67)
Age at Diagnosis	0.58	(0.50, 0.66)
Sex	0.56	(0.49, 0.63)
BMI	0.55	(0.44, 0.64)
Pack per year	0.55	(0.49, 0.62)
Phenotype (non-ComBat)	0.48	(0.47, 0.59)

**Table 4:** Multivariate analysis results. A baseline model based on ECOG + Age significantly improves (p=0.003 ) when the clinical variables of the baseline model are integrated with the imaging phenotype obtained from harmonized features using ComBat, i.e., Phenotype (ComBat) + ECOG + Age.

Predictor	C-Score	95% CI	*p*-Value (LRT) *
Phenotype (ComBat) + ECOG + Age	0.69	(0.62, 0.77)	0.003
Phenotype (non-ComBat) + ECOG + Age	0.66	(0.58, 0.74)	0.15
ECOG + Age (Baseline)	0.65	(0.57, 0.73)	--
2 PCs + ECOG + Age	0.65	(0.60, 0.75)	0.27

* *p*-value of LRT with respect to the baseline model (ECOG + Age).

## Data Availability

Data available on request due to restrictions, e.g., privacy or ethical. The data presented in this study are available on request from the corresponding author.

## Supplementary Materials

The following are available online at https://www.mdpi.com/article/10.3390/cancers14030700/s1. Figure S1: Kaplan–Meier curves for univariate analysis; Figure S2: Statistical differences of continuous covariates among clusters defined by the imaging phenotype based on harmonized features; Figure S3: Kaplan–Meier curves for multivariate analysis; Table S1: Assessment of the imaging phenotype based on harmonized features. Sample codes implemented in R are also available online at https://github.com/jmlunac/phenotype-nsclc-survival (accessed on 21 January 2022).
