# Supplementary material for: Radiomic Phenotypes for Improving Early Prediction of Survival in Stage III Non-Small Cell Lung Cancer Adenocarcinoma after Chemoradiation
Source: Cancers (Basel). 2022 Jan 29;14(3):700. doi: 10.3390/cancers14030700 (PMC8833400; doi:10.3390/cancers14030700)
Supplement: Supplementary file 1 [file cancers-14-00700-s001.zip › cancers-1486324-supplementary.pdf]

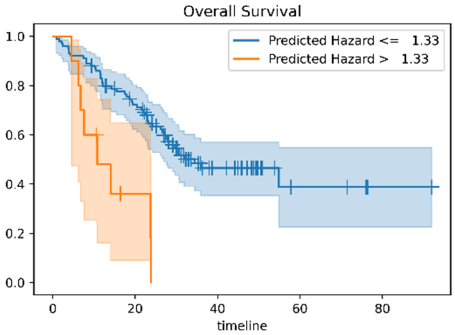

(**a**) ECOG

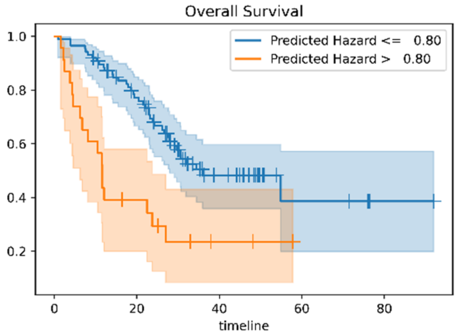

(**b**) Phenotype (ComBat)

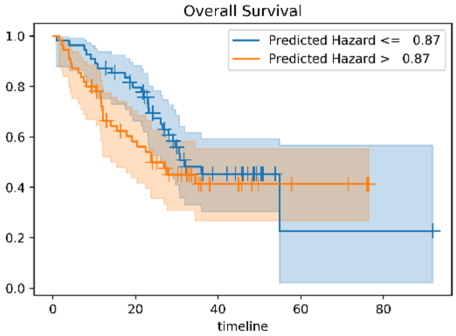

(**c**) Tumor Volume

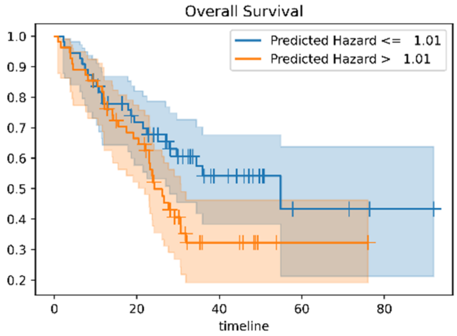

(**d**) Age

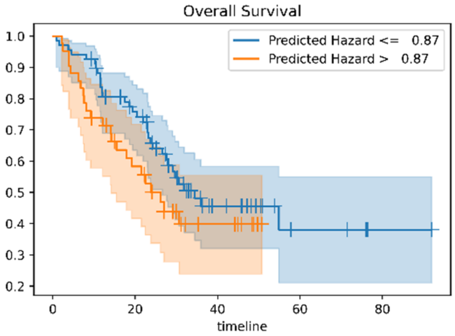

(**e**) Sex

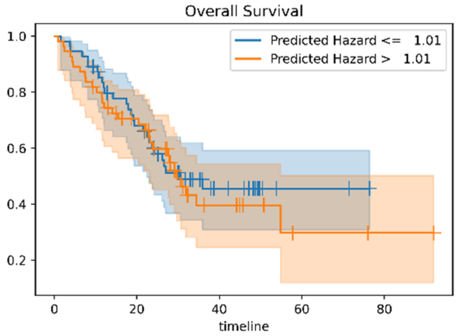

(**f**) BMI

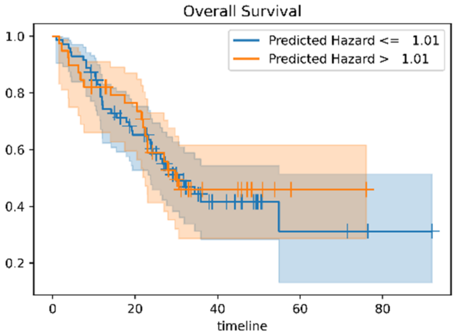

(**g**) Pack-Year

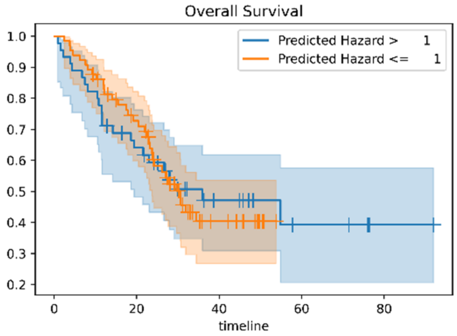

(**h**) Phenotype (non-ComBat)

Figure S1. Kaplan-Meier curves for Univariate Analysis. The only variables able to predict systematic survival differences in our cohort are (**a**) ECOG (C-score = 0.62, 95% CI [0.55,0.69]), and (**b**) Phenotype (ComBat) (C-score = 0.61, 95% CI [0.54,0.67]). The remaining variables, i.e., (**c**) Tumor Volume (**d**) Age, (**e**) Sex, (**f**) BMI, (**g**) Pack-year, and (**h**) Phenotype (non-Combat) do not show good predictive performance.

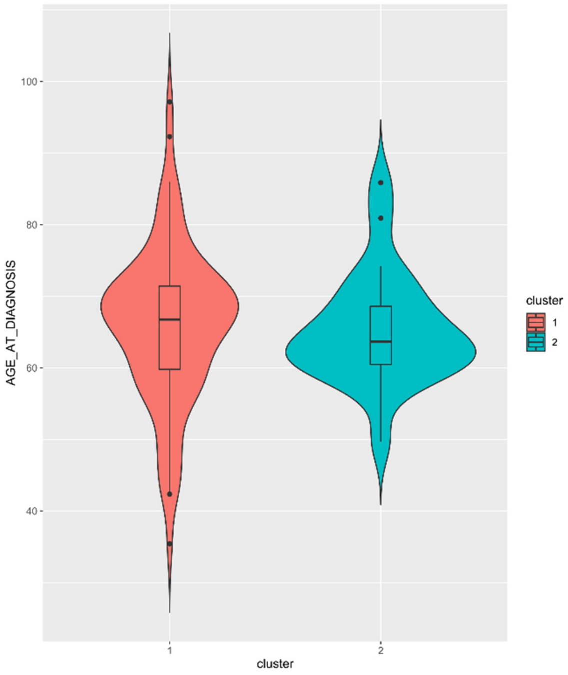

(**a**) Age

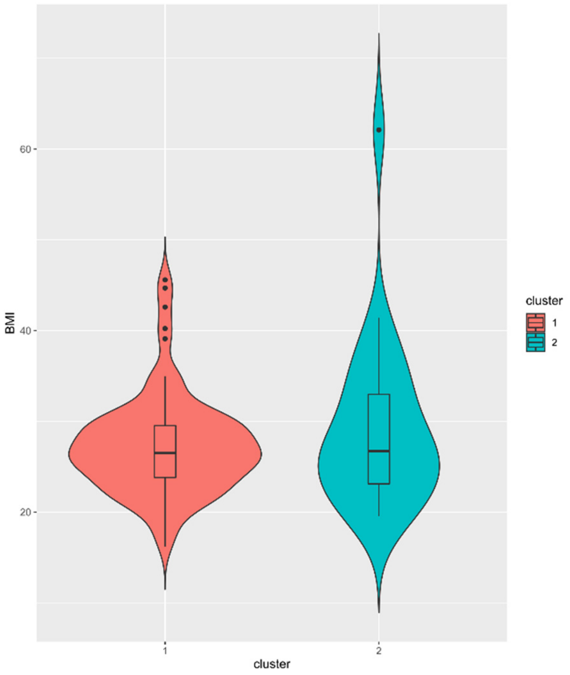

(**b**) BMI

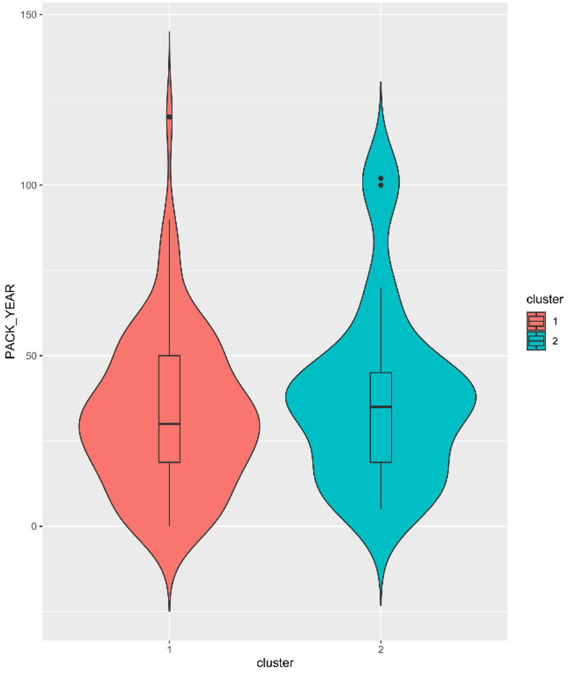

(c) Pack-year

Figure S2. Statistical differences of continuous covariates among clusters defined by the imaging phenotype based on harmonized features. Violin plots illustrating statistically significant differences of the continuous clinical covariates among the clusters identified by the Phenotype (ComBat) model. Notice that based on the Kruskal-Wallis test, there are no significant differences between the two clusters defined by the phenotype in the selected continuous clinical covariates, i.e., (**a**) Age (p = 0.42), (**b**) BMI (p = 0.58) and **(c)** pack-year (p = 0.42).

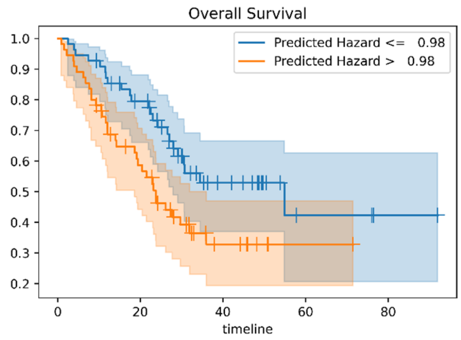

(**a**) ECOG+Age (Baseline)

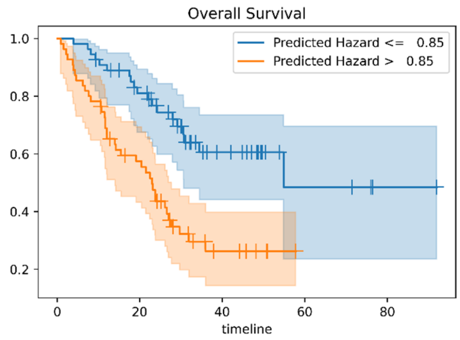

(**b**) Phenotype (ComBat)+ECOG+Age

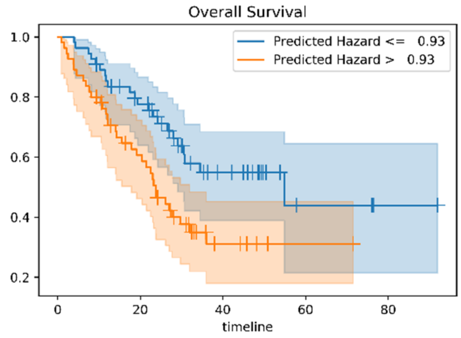

(c) Tumor Volume+ECOG+Age

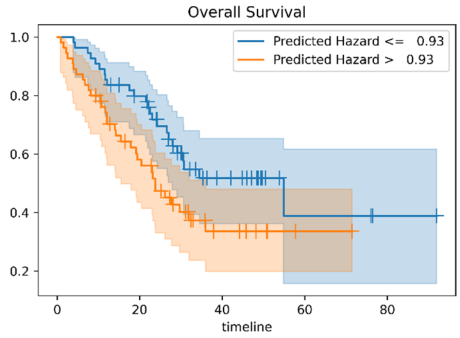

(d) 2 PCs +ECOG+Age

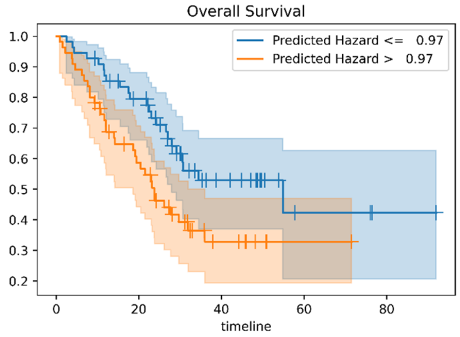

(e) Phenotype (non-ComBat)+ECOG+Age

Figure S3. Kaplan-Meier curves for Multivariate Analysis. Kaplan-Meier curves illustrating the non-cross-validated predictive performance of (**a**) The ECOG + Age model (C-score = 0.65, 95% CI [0.57,0.73]), (**b**) Phenotype (ComBat) + ECOG + Age (C-score = 0.69, 95% CI [0.62,0.77]) and (**c**) Tumor Volume + ECOG + Age (C-score = 0.66, 95% CI [0.59,0.75]). The improvement of the models integrating the imaging phenotype and the model integrating tumor volume with clinical variables were both significant with LRT $p = 0.003$ and $p = 0.03$ respectively. However, neither the **(d)** 2 PCs + ECOG + Age model (C-score = 0.65, 95% CI [0.60,0.75]) nor the **(e)** Phenotype (non-ComBat) + ECOG + Age (C-score = 0.66, 95% CI [0.58,0.74]) models exhibited a significant improvement with LRT $p = 0.27$ and LRT $p = 0.15$ respectively. Finally, **(c)** Tumor Volume + ECOG + Age performs significantly lower ($p < 0.005$) than (**b**) Phenotype (ComBat) + ECOG + Age.

Table S1. Assessment of the imaging phenotype based on harmonized features. Assessment of statistically significant differences of relevant clinical covariates among the clusters identified by Phenotype (ComBat). Notice that ECOG is the only covariate that the phenotype can predict ($p = 0.048$). Also, Phenotype (ComBat) is able to predict ($p = 0.025$) the event of death, (i.e., alive v. dead).

**Categorical Covariates**	**Kruskal-Wallis Test (*p*-value)**
Death	0.025
ECOG	0.048
Race	0.341
Progression	0.363
Sex	0.892
**Continuous Covariates**	**Chi-squared test (*p*-value)**
Pack-year	0.417

Age	0.421
BMI	0.581

Sample codes:

Sample codes implemented in R are also available online at https://github.com/jmlunac/phenotype-nsclc-survival
